# Collectanea Medica

**Published:** 1814-02

**Authors:** 


### COLLECTANEA MEDICA,

CONSISTING OF

ANECDOTES, FACTS, EXTRACTS, ILLUSTRATIONS, QUERIES, SUGGESTIONS, &c.

RELATING TO THE

*History or the Art of Medicine, and the Auxiliary Sciences.*

### *Vaccination.*

MR. RIGBY, of Norwich, has printed an interesing Report of the Pauper Vaccination in that city, from August 10th, 1812, to August 12th, 1813. The plan which he suggested was, that each individual vaccinated should receive half-a-crown from the Court of Guardians of the Poor. The vaccination commenced immediately, and has been continued with the most important result to humanity. Two thousand three hundred and ninety-one individuals were vaccinated in the course of one year, between the 10th of August, 1812, and the 10th of August, 1813. Of these, 1508 have each received half-a-crown, offered by the Court of Guardians, amounting to the sum of 188l. 10s.

Since the introduction of vaccination, there has been no instance, in this country at least, where, in a place of the like population, so many poor persons have, within the same

 period period of time, received the benefit of this inestimable discovery; and certainly there has been no instance in England, in which so many have been vaccinated at so small an expence to the public. We wish this important fact may be generally known, that other places may profit by the example of Norwich, and adopt the same means for the diminution of human misery, and for the preservation of human life.

*Report.*—Having, with every friend to humanity, lamented the difficulty of extending the benefit of vaccination to the lower classes of society, which, for many years, have, in this country, been the principal sufferers from small-pox, and having found the exertions of individuals, and even the establishment of societies, one of which, a few years ago, was formed in this city, unavailing, sufficiently to interest the poor on this important subject, I considered it indispensable, in any attempt to effect an extensive pauper vaccination, to profit of the influence and authority of those bodies of men to whom, in most large cities, the law has peculiarly entrusted the care of the poor; and, at the same time, to endeavor to obtain the consent of the poor to such a measure, by holding out to them the additional inducement of a reward.

In this city the important duty of taking care of the poor rests with sixty citizens, called Guardians of the Poor, and who hold a monthly court; some of these are magistrates, and being myself, as a magistrate, a member of this court, I have several times urged it to take this subject into consideration.

In the beginning of August, 1812, having information that the small-pox had appeared in some neighbouring villages, I was induced to renew my earnest application to the court on the subject, and I succeeded in obtaining not only its sanction for a general gratuitous vaccination of the poor, but its concurrence in my proposal that each individual vaccinated should receive half-a-crown. This resolution of the court was publicly notified, and the three city surgeons were directed to vaccinate all poor persons, resident in the city, who should apply, whether they, parochially, belonged to it or not.

The vaccination commenced immediately, and the readiness with which the poor submitted to it is manifest, from 754 persons, principally children, having been vaccinated from the 10th of August to the 10th of September. It has been continued from that time to the present, with the exception only of the month of January, when all alarm respecting small-pox had subsided; and the result has been most important to humanity, 2391 individuals having, in consequence of this measure, had the benefit of vaccination, between the 10th of August, 1812, and the 10th of August, 1813; which is, probably, a greater number than, within the same period, have been vaccinated in any other place, in this country, of like population.

Of these 1508 have each received half-a-crown, the court having paid the sum of 188l. 10s. for this purpose.

As before observed, the vaccination was first had recourse to in consequence of the near approach of the small-pox. The promptness with which it was undertaken, and the considerable numbers which were immediately vaccinated, seem effectually, at that time, to have prevented its spreading in the city, though a case of natural small-pox, traced from a neighbouring village, had occurred, and an additional source of infection had subsequently been introduced by variolous inoculation, for until the month of February not a single case of the disease existed, at which time 1415 had been vaccinated.

In the beginning of February a soldier's wife, who had passed through London, with three children, came into the city—her eldest boy was full of the small-pox, and the two other children were sickening with it, all of them having caught it in London. This unfortunate fact was soon made public, and the vaccination immediately again had recourse to; but the small-pox, on this occasion, soon found its way among the unvaccinated, and several children were sacrificed to it within a few weeks; for it had appeared, nearly at the same time, in a different part of the city, and it was ascertained to have been, also, brought thither by another unfortunate communication with the metropolis. The number of persons who had not profited of the vaccination in the preceding autumn, proved now to be greater than was expected; for calculating on the probable average number of annual births in the lower classes, and on the supposed numbers of individuals who had the small-pox in the years 1808 and 1809, I had estimated the number liable to the disease, in the beginning of August, 1812, not to have exceeded 1300.

Though many of the poor were daily vaccinated, more, indeed, than I still calculated to be liable to the small-pox, there was an unfortunate number who yet neglected the boon, and among these the disease has, from that time to the present, spread itself, and I regret to record that, from the 10th of February to the 3d of September, 65 deaths have occurred from it. How much greater the sacrifice of human life would have been, had not so extensive a vaccination taken place, may easily be conjectured—had those, who were vaccinated, been liable to take the infection, the deaths would, probably, have exceeded 400.

During this time, I am sorry to say, there were many instances of the grossest carelessness in the exposure of patients, in all stages of the disease, in the most public streets. It was also admitted into several public-houses, the resort of country persons, and thence communicated to some neighbouring villages. I failed, in an application to the acting magistrate, to take some steps to ascertain what public-houses were thus infected, and to guard strangers, liable to the disease, against entering them, as he conceived there was no law to countenance such an interference.

The vaccination, in the preceding autumn, having been so completely successful in preventing the spread of the small-pox, was unquestionably a most gratifying circumstance; but though during the subsequent vaccination, from February to August, the disease still made a fatal progress, the melancholy fact has afforded an irrefragable proof of the protecting power of vaccination: during this period period probably not fewer than 400 individuals have had the small-pox; there has likewise been no intermission of the disease—it has been constantly spreading, and on many occasions, patients, as before observed, have been publicly exposed. Of the 2391 vaccinated during the year, it may be assumed, that at least 2000 have been resident in the city since February, and consequently equally exposed to an infectious atmosphere as the unvaccinated, and yet but one single instance, in that number, has occurred, in which the protecting influence of vaccination has been suspected, and this has been clearly ascertained to have been a case of premature vesicle, which suddenly rose, soon disappeared, and evidently produced no constitutional affection.* Whereas every unvaccinated person, thus exposed, has probably taken the disease, and I should fear, of those remaining at present uninfected, if not promptly vaccinated, that very few will escape.

From the intercourse between Norwich and the neighbouring villages, the small-pox was soon introduced into them, and has since spread into various parts of the country: it has also prevailed much, and been very fatal at Yarmouth, where few have been vaccinated; many of the local militia were infected there, and communicated it, on their return, to their respective villages. Its progress has also been much accelerated, and its diffusion promoted, by the unjustifiable, and much to be reprobated practice of variolous inoculation. I lament to say, that some professional men have allowed themselves to inoculate for small-pox; but it has been principally done by despicable empirics, itinerants, even shoe-makers, and old women; and in some instances, it has been ordered by ignorant overseers of parishes.

In such a disastrous state of the disease, spreading, as it is, at this time, through many populous districts, a general gratuitous vaccination of the poor cannot be too much urged. The signal success of the measure in Norwich has at once established its practicability, and shown that the means of effecting it are within the reach of every parish in the kingdom. That these means are not of difficult application is evident from its having been there carried through without the smallest agitation of the public mind—without any interruption

* This was a child of Mrs. Gostling, in Grant's Yard, St. John's of Madder Market; it had been vaccinated in April; I visited it on the 29th of July: there were many recent vestiges of distinct variolæ upon it. On examining the vaccinated arm, I could detect no cicatrix, and Mrs. Gostling, unasked, said she attributed the failure to the vesicle having risen sooner, subsided sooner, and having been less than those in her other three children, who were vaccinated at the same time, and who have all resisted the infection.

As a singular coincidence, I would observe, that about this time my friend Mr. Chandler, of St. Faith's, called upon me to say that he had just seen a patient under small-pox, who had been inoculated by him, for that disease, nine years ago, and then appeared to have had the genuine small-pox.

to public business—with even but little loss of time to the poor themselves—and comparatively with little professional inconvenience. And that it is not objectionable on account of the parochial expence incurred by it, is equally evident, from 2391 having been vaccinated at an expence to the city of only 188l. 10s., and against this must be placed the expence which would have accrued to the city, had even half that number taken the small-pox. The vaccination of eight in a family, each taking the reward, brings an expence of only 1l.; but should four only in a family sicken with this disease, an expence to the amount of several pounds would, probably, be incurred. The derangement of a poor family, by the introduction of small-pox, is of the most distressing kind; an immediate suspension of labour takes place; the time and attention of every one are directed to the poor sufferers; increased wants arise; the necessaries for the sick are of a more expensive kind; the means of supplying them cease; the wretched father can earn nothing, he has neither time nor ability to work—he is compelled to apply for parochial relief; and though parishes often give with too sparing a hand, on no occasions have parochial burdens been more severely felt, than when the small-pox has spread through a district.

It has been objected to this measure, that medical men cannot be expected always to vaccinate the poor without some remuneration. In all places of considerable population, the medical care of the poor is contracted for, at a fixed annual stipend; and though I fear, there are few instances of this kind, in which surgeons are paid with adequate liberality, yet when a parish, or a district, is taken, it becomes an object of economy and policy to vaccinate every pauper, rather than risk the expence, trouble, and I may certainly add, the anxiety of attending them under so horrid a disease. On this ground, I am persuaded, there is nothing to fear. There has been, comparatively, little reluctance shewn by medical men, to extend the great benefits of the Jennerian discovery; on the contrary, with few exceptions, much good sense has been evinced in their early appreciation of its high value, and much zeal and personal exertion have been used by them, to establish and extend its beneficial influence in society.

But still more effectually to prevent the future admission of small-pox, and to increase the probability of its ultimate extinction, an event which every friend of humanity will indulge in anticipating, more is unquestionably necessary. It has been ascertained, that small-pox inoculation is the great means by which the disease is kept in existence,* and that London, through its agency, is the great generating focus of variolous infection, whence, as in its late communication to Norwich, it radiates to every part of the empire. While this practice continues, it will be utterly impossible to extinguish small-pox, or to prevent the occasional alarm even of those who have been vaccinated. It is therefore indispensable to the interests of humanity, that the practice should cease; but it is pretty evident, while

* Report of the National Vaccine Establishment, 1813.

any any pecuniary gain attaches to it, individuals will be found to practise it; and while any prejudices remain against vaccination, which it is their obvious interest to keep up and increase, there will be no difficulty in finding subjects to practise upon.

Under these circumstances there appears no resource but in legislation; and the accumulated mischiefs which have already resulted from the practice, together with its direct tendency to destroy life, by diffusing a pestilential and dangerous disease, will surely justify the passing a law, imposing a severe penalty on any one, directly or indirectly concerned in the act of variolous inoculation.

The greater number of those who are agents in this mischievous practice, are, I am persuaded, incapable of reasoning on the subject, and probably still less capable of any moral discussion; but those few of the profession, who continue it, assume, as a justification, the alleged insecurity of vaccination; and to establish such an opinion, are loud in their abuse of it, industrious in collecting cases of doubt or error in vaccination, some of which must necessarily occur in very great numbers, in magnifying supposed failures, and in laying them most conspicuously before the public in newspapers, &c.

Though most of these cases of imputed failure have been satisfactorily explained,* an unfavorable impression has, certainly, been made on the public mind, the progress of vaccination has been impeded, and what is to be lamented, doubts and alarms have been excited in the minds of those who have been vaccinated; on this account, I have no hesitation in saying, the opposers to vaccination have much to answer for. Admitting their adduced cases of failure to their utmost extent, vaccination would still be a blessing; it would be no reason for abandoning vaccination, but a powerful one for preventing the introduction of small-pox, and for increasing our efforts to exterminate it; and assuming it possible that the failures were even one in twenty, the means of effecting this important purpose would, obviously, be still increased in the proportion of nineteen to one. I will say further, and say it gravely, that, on a fair comparison of the two inoculations, no man of sound intellect, of correct moral feeling, and who does not prefer his individual interest to the general and more important interests of society, will hesitate to prefer vaccination to variolation. When no other means of lessening the fatality of small-pox than inoculation were known, the practice was justifiable; and had the security which individuals obtained from it, been extended to all classes of society, and no injury done to any, it would have been a general blessing. It is well known, however, that its benefits were ever limited to certain orders, and that the poor never having been inoculated to any extent, not only failed to profit of it, but the disease being constantly kept up by a never-ceasing partial inoculation of their richer neighbours, they were, more than ever, exposed to infection, and the average mortality from it, though almost exclusively confined to them, became even greater than before

* See Norfolk Chronicle, Sept. 4, 1813.

the discovery of inoculation; population proportionately suffering. With vaccination this could not have been—the protection it gives is without alloy; with it, the security of one is not obtained by exposing others to danger: it is not an infectious disease.

Inoculated small-pox is attended with illness in its eruptive stage, sometimes with severe symptoms in every stage, and it has been calculated to have occasioned death in the proportion of 1 in 300.

A very few years ago, indeed, inoculated small-pox was much more fatal, in several parts of Norfolk—at Elmham, Holt, and Aylsham. In Aylsham, considerable numbers were inoculated, and the deaths were nearly 1 in 20.

The peculiar constitutional change induced by small-pox, by which scrofula has been so often elicited, is not confined to the natural disease; the inoculated small-pox, when severe, has the same prejudicial influence on the constitution, and this has been one of the popular objections to it. I have only one other observation to make—variolous inoculation does not, in every instance, prevent the future admission of the disease;—there are many well-attested cases of its recurrence.

Vaccination, on the contrary, through its whole progress, is attended with very little perceptible illness, there is no eruptive fever, there are no frightful convulsions in children, and as it does not in any degree disturb the system, it cannot be charged with exciting any latent morbid affection; it is wholly free from danger; and notwithstanding the magnified reports of the anti-vaccinists, the instances of its failing to secure against small-pox, are not more than those which have followed variolous inoculation.

From a report to the French government on the general state of vaccination, it appears that the proportion of failures in France has been only as 1 to 381,666.*

In all the reflecting and educated classes, there is little doubt of vaccination being generally adopted. But I am advocating the vaccination of the poor. With them, the means of information, on important subjects, are not very attainable; nor can they, for want of intellectual improvement, readily appreciate new discoveries, at all connected with science. On all subjects involving the happiness of the lower classes, much is expected, from the exerted influence of the higher and more educared classes;—and on this, it would seem their obvious duty, to give them information, to remove their doubts and prejudices, and to offer motives for their consent to a measure of such importance, not only to their individual welfare, but to the general interests of society. I lament to say, little disposition of this kind has shown itself; and from what I have observed, I fear it will be difficult to excite among the higher classes such an interest on this subject, as to induce, on their part, any extensive exertion on behalf of pauper vaccination.

The efficiency of a gift offered to the poor, has been unequivo-

* National Vaccine Establishment for 1813.

cally proved in the late extensive pauper vaccination in Norwich, and there is little doubt but a similar measure would be equally successful in other places: but the truth must not be concealed, there appears no disposition to follow this example. Even in the immediate neighbourhood of Norwich it has not been adopted, nor has the experiment been attempted in any part of the county of Norfolk. I fear, therefore, that even this measure, which promises so effectually to establish vaccination among the poor, will not be generally, if at all, had recourse to, unless enjoined by legislative authority—and if considered as a parochial allowance, there would seem no more objection to parliament directing a reward, on the vaccination of the poor, to be paid in every parish in the empire, than there is in its directing a specific allowance to the wives and children of militia-men.

Under a conviction, therefore, that the destructive progress of the small-pox cannot be arrested, unless variolous inoculation be completely put down; and that no measure is so calculated to produce the general vaccination of the poor, (which seems equally necessary to the extinction of small-pox,) as the giving to each person vaccinated a small pecuniary reward; and being not less satisfied that neither of these measures can be carried into any extensive effect, without legislative interference, I trust the subject, important as it is in a moral, and in a national view, will, in due time, engage the attention of parliament.

*Norwich, Sept.* 6, 1813.

